# Orthorexia nervosa, eating patterns and personality traits: a cross-cultural comparison of Italian, Polish and Spanish university students

**DOI:** 10.1186/s12888-019-2208-2

**Published:** 2019-07-30

**Authors:** Carla Gramaglia, Eleonora Gambaro, Claudia Delicato, Marco Marchetti, Marco Sarchiapone, Daniela Ferrante, María Roncero, Conxa Perpiñá, Anna Brytek-Matera, Ewa Wojtyna, Patrizia Zeppegno

**Affiliations:** 1 Psychiatry Ward, Maggiore della Carità University Hospital, Novara, Italy; 2 Department of Translational Medicine, Institute of Psychiatry, Università del Piemonte Orientale, Novara, Italy; 3 Department of Medicine and Health Sciences, University of Molise, Via DeSantis, 86100 Campobasso, Italy; 4 Departamento de Personalidad, Evaluación y Tratamientos Psicológicos. Facultad de Psicología, Universitat de València. Av. Blasco Ibáñez, 21. 46010 Valencia, Spain; 5 CIBER Fisiopatología Obesidad y Nutrición (CIBERobn), Instituto Salud Carlos III, Madrid, Spain; 6 Katowice Faculty of Psychology, SWPS University of Social Sciences and Humanities, Katowice, Poland; 7 Institute of Psychology, University of Silesia, Katowice, Poland

**Keywords:** Orthorexia nervosa, Eating patterns, Personality traits, Cross-cultural comparison

## Abstract

**Background:**

The amount of research about orthorexic attitudes and behaviours has increased in the last five years, but is still mainly based on descriptive and anecdotal data, yielding a variety of prevalence data and inconsistent results. The interplay between socio-cultural context and orthorexia has been poorly investigated and is still far from being understood.

**Method:**

Multicentre, cross-sectional study involving Italian (*N* = 216), Polish (*N* = 206) and Spanish (*N* = 242) university students, assessed through a protocol including informed consent, socio-demographic and anamnestic data sheet and self-administered questionnaires (ORTO-15, Eating Attitudes Test- 26 [EAT-26], Temperament and Character Inventory [TCI]).

**Results:**

Higher prevalence of orthorexia (as described by the ORTO-15 cutoff) was found in Poland. Female gender, Body Mass Index (BMI), current Eating Disorder, dieting, EAT-26 score ≥ 20 and low/medium Persistence were associated with orthorexia in the whole sample. The cross-cultural comparison showed several differences among the three subgroups of students.

**Conclusions:**

The associations found between orthorexic attitudes, self-reported current eating disorder, BMI and adherence to a dieting need to be supported by further research. The differences among students from the three countries seem to suggest a possible rolve for cultural elements in the construct of orthorexia.

## Highlights

- The interplay between socio-cultural context and orthorexia has been poorly investigated and still needs to be better understood.
- The associations found between orthorexic attitudes, self-reported current eating disorder, BMI and dieting may be explained by the great attention paid to the individual responsibility for health, typical of Western societies.
- Differences in the frequency of orthorexic attitudes and behaviors among the Italian, Polish and Spanish samples can be related to the role of gastronomic culture, Mediterranean diet, convivial and social value attributed to eating and main approaches aimed at improving one’s health.

## Background

Eating styles and behaviors have always been and are still currently deeply shaped by the context where people live, encompassing cultural, social and environmental factors [1]. For instance, Western societies have long been influenced by theoretical constructs on the purity of the body obtained through food restrictions, in a mind over matter framework that extends from classical culture and early Christian beliefs to contemporaneity [2].

In contemporary society, the interest in the body and the will to keep it healthy have become “obsessive” and sometimes leading to eating behaviors based on the compulsive search for natural and “pure” foods [3]. Recently, a new health consciousness", called “healthism” [4] suggests that health can be accomplished in an unproblematic way through individual discipline and conduct, mainly by doing regular exercise and healthy eating [5]. Further context-related elements which have gained importance and become an important part of this phenomena over the last years [6, 7] include the mass-media and the Internet.

The concept of orthorexia nervosa (ON) defined as as a persistent fixation on healthy eating [8] has developed in this cultural and temporal context. Currently, though ON is not classified as a formal diagnostic category and its grading is still a matter of debate [9], several suggestions have been made about it being a distinct subtype of the Avoidant/Restrictive Food Intake Disorder (ARFID) described by the Diagnostic and Statistical Manual of Mental Disorders [10], sharing features with anorexia nervosa (AN) [11] or overlapping with obsessive-compulsive disorder [2]. While Dunn and Bratman [8] pointed out that ON could be considered as a distinct condition, the current state of research does not allow the final categorization of ON as a separate mental disorder [12]. Actually, despite the increase in the amount of research about this topic in the last five years [11, 13], the literature on ON is still mainly represented by descriptive and anecdotal data, often with inconsistent results [14], and its prevalence in the general population has been recently estimated to be less than 1% [8].

Regarding the shared features between ON and AN, there are cognitive fixation on nutrition, perfectionism, high anxiety and need to exert control, achievement orientation, guilt over food transgressions, value adherence to dieting as a marker of self-discipline, limited insight, cognitive rigidity, denial of the functional impairments associated with disorder [9]. Therefore, it is likely, but not yet consistently supported by the existing literature, that some AN features, including personality traits, could be involved in the development and maintenance of ON as well [12, 15].

The personality correlates of ON have been addressed only by a few studies. Higher levels of neuroticism [16], narcissism and perfectionism [17] were described in individuals with ON -related tendencies, cognitions, behaviors, and feelings. A recent study [18], performed with a widely used assessment tool for personality, i.e. the Temperament and Character Inventory (TCI) [19], has investigated personality traits in ON individuals, showing high Harm Avoidance, high Self-Trascendence and low Self-Directedness. According to these dimensions, an “orthorexic personality” has been described as characterized by excessive preoccupation and shyness in social situations, combined with the desire to be perfect and to feel accepted [18]. Further conclusions of this study suggested that strictly planned, excessively “pure” dieting would result from an intense need for control and as a compensation of poor self-esteem and feelings of ineffectiveness in managing harmful events [18].

According to these premises, we identified three main areas of interest: 1) a deeper understanding of the orthorexia construct, considering its possible and currently debated relation with full-blown eating disorders; 2) the investigation of the personality correlates of orthorexic attitudes and behaviors; 3) the role of the cultural context on orthorexia.Regarding the first area, there is still much inconsistency in the existing literature. A previous study [20] by our research group investigated orthorexia in a sample of patients with a clinical diagnosis of eating disorders, showing that more pathological ON attitudes and behaviors (as measured by the ORTO-15 test) were related to lower degrees of disordered eating (as assessed with the Eating Attitudes Test-26). While another study has supported a negative correlation between ON and disordered eating patterns [21], others (using the Bratman Orthorexia Test and the Eating Habits Questionnaire, respectively) found increased ON features corresponding to more severely disordered eating behaviors (particularly calories restriction and weight concerns) [2].

As far as personality is concerned, considering its role in eating disorders [22], the widespread use of the TCI and the familiarity we have with this assessment tool [23, 24], we decided to include it in our research.

Last, the interplay between socio-cultural context and ON has been poorly investigated. We already addressed this topic in a previous study [20] involving Italian and Polish females with AN and healthy controls. Less orthorexic features, as assessed with the ORTO-15, emerged in the Italian than in the Polish samples (both AN and healthy control group). Research involving non-clinical populations of students have reported a wide variability in the prevalence of orthorexic attutides and behaviors, being up to 68.55% in Polish University students [26], and57.6% in a previous Italian study [25].

Therefore, the aim of the present study was: 1) to assess the prevalence of orthorexic attitudes and behaviours (as measured by the ORTO test; later on referred to in the text as ON, for the sake of conciseness) in a sample of university students enrolled in different European countries (Italy, Poland and Spain); 2) to investigate the association between ON, socio-demographic features, eating patterns (measured with the EAT-26) and personality traits (assessed with the TCI) within the whole sample; and 3) to investigate the possible cross-cultural differences in ON and eating patterns in the three samples, subdivided according to nationality.

We put forward the hypothesis that ON prevalence and the differences in eating patterns could reflect eating habits in Italy, Poland and Spain as a mirror of cultural normative pressures.

## Methods

### Study design

Multicentre, cross-sectional study analyzing the ON construct, eating habits and personality traits in a sample of European university students from Italy, Poland and Spain. We recruited a convenience sample, based on previous collaboration among some of the authors (P.Z. and C.G., A.B.M., M.R.). Each of these authors proposed the research protocol to the students attending their University courses (different types of university courses were involved, as detailed below, thus ensuring a good representativity of the University population), whowere assessed with a protocol including informed consent, socio-demographic and anamnestic data sheet and self-administered questionnaires.

### Sample

The sample was composed by Italian, Polish and Spanish university students enrolled by the Psychiatry Institute, Department of Translational Medicine, Università del Piemonte Orientale at the Faculty of Medicine and Nursing Sciences, in Novara, Italy; by the Psychology Faculty of Katowice at different universities in Silesia, Lower Silesia, Lesser Poland and Mazovia regions (philosophy, dietetics, psychology, sociology students) in Poland; and by psychology students from the Faculty of Ciencias Sociales y Humanas of the Universidad de Zaragoza, in Teruel, Spain, and from the Faculty of Psycholgy, Universitat de València, in Valencia, Spain. Recruitment took place from January 1st 2016 to December 31st 2017. No inclusion/exclusion criteria were applied. Students were approached during lesson time (hence it was not possible to retrieve the exact number of those approached, nor that of those who declined); they were explained the study protocol and asked about their willingness to participate and to provide their informed written consent. In case they accepted to take part in the study, they were e-mailed a link with the study protocol, allowing anonymous compilation. The data sheet gathered information about gender, age, ethnicity, marital status, weight and height (in order to allow the calculation of BMI), use of substances and/or alcohol, cigarette smoking, sporting activity, dieting, use of nutritional supplements and past or current ED diagnosis. All the information gathered was self-reported, including that about anthropometric measures and ED diagnosis.

### Assessment

Participants were assessed with the following questionnaires: ORTO-15 [26, 27]; Eating Attitudes Test-26 (EAT-26) [28]; Temperament and Character Inventory (TCI) [19, 29].

#### Orto-15

The ORTO-15 is a self-administered scale developed in 2004 by Donini and coworkers, modeled on the Bratman test [30, 31]. It is the most frequently used tool for the assessment of orthorexic symptoms in various populations [26, 32, 33], even though it is clearly not a diagnostic tool.

The ORTO-15 was developed in Italian [26] and later translated in other language. Several studies have eliminated one or more of its items, based on confirmatory analyzes and the adequacy of the instrument [34].

ORTO-15 scores range between a minimum of 15 and a maximum of 60, with lower scores corresponding to more pathological behaviors. Two cut-offs for orthorexic behaviors have been proposed: < 40 (sensitivity 100%, specificity 73.6%, positive predictive value 17.6%, negative predictive value 100%) and < 35 (sensitivity 86.5%, specificity 94.2%, negative predictive value 94.1%) [28, 35].

The Polish version of the ORTO-15 has a maximum score of 36 and adopts a 24-points threshold cut-off [25].

Since our study involved samples recruited in Italy, Poland and Spain, the cutoff of 35 was used for Italy and Spain, and the cutoff of 24 for Poland, to identify possible ON, as described in the literature [36]: therefore, students were classified dichothomically, as scoring under or above the specific cutoff scores.

### Eating attitudes Test-26 (EAT-26)

The EAT-26 is the most common self-report measure assessing symptoms and concerns typical of EDs [27]. The scale consists of 26 items and includes three basic dimensions: dieting (which reflects restricting intake of high caloric foods and preoccupation with body image/shape), bulimia and food preoccupation (which describes thoughts regarding food, binging and self-induced vomiting) and oral control (which is about the ability to regulate food intake and perceived pressure from others to gain weight). Albeit it is not a diagnostic tool, it has been suggested that the EAT-26 might identify cases at risk for EDs clinical spectrum [37]. A score of 20 or higher on the EAT-26 indicates a high level of concern about dieting, body weight or problematic eating behaviors. The questionnaire has been validated and translated into Italian [37], Polish [38], and Spanish [39] and used extensively in clinical settings as well as in the general population.

### Temperament and character inventory (TCI)

Developed by Cloninger [19], it is a self-report scale consisting of 240 questions with yes/no answers. It is based on the biopsychosocial personality theory developed by Cloninger and assesses individual differences in the seven basic dimensions of Temperament and Character, including four temperamental dimensions (Novelty Seeking [NS] mediated by dopamine; Harm Avoidance [HA] mediated by serotonin; Reward Dependence [RD] mainly mediated by norepinephrine; Persistence [P] mediated by glutamate) and three character dimensions (Self-Directedness [SD], Cooperativeness [C], Self-transcendence [ST]). The Temperament and Character Inventory-Revised (TCI-R) [29] allows graded answers from 1 (absolutely false) to 5 (absolutely true) on a Likert scale. The questionnaire has been validated and translated into Italian [40], Polish and Spanish [28] and used extensively in clinical settings as well as in the general population. Due to availability reasons, the TCI was used in Italy and Poland, while the TCI-R was used in Spain. To overcome the possible problems entailed by the different scoring methods of the scales used, we adopted the cutoffs described in the literature [19]. Scores in each temperament and character dimension were thus classified accordingly as low (low/very low scores); medium (medium-low/medium/medium-high scores); high (high/very high scores).

### Ethics

The study was approved by the local ethics committees (Comitato Etico Interaziendale, Novara, Italy, protocol UNI-ORTO, no. 1/2014; University of Silesia in Katowice Human Research Ethics Committee, no. 14/2015; Comité Ético de Investigación en Humanos de la Universitat de València, no. H1409824786250). All procedures performed in the study were in accordance with The Code of Ethics of the World Medical Association (Declaration of Helsinki). Participation was anonymous and informed written consent was obtained from participants. No fee or reimbursement was offered for participation in the study.

### Statistical analyses

To quantify categorical variables, frequency distribution tables were constructed for categorical variables and mean and standard deviation were calculated for continuous variables.

Univariable and multivariable logistic regression models were used to study the association between the study variables and the presence of ON (as measured by an ORTO-15 score below the cutoff). Odds ratios and 95% confidence intervals (95% CI) were calculated. The significance of each individual variable was assessed using the likelihood ratio test (LRT). The Kruskal-Wallis test was used to evaluate the difference in continuous variables between the groups of students from Italy, Poland and Spain and for categorical variables the association with the nationality was evaluated by the chi-square test or Fisher test.

A *p*-value < 0.05 was considered statistically significant. Statistical analyses were performed with STATA v14.

## Results

### Descriptive analysis

We enrolled 664 university students, 216 in the Italian University, 206 in the Polish University, 242 in the Spain University. The percentage of females was 72.29%, and the sample mean age was 24.02 years (SD: 4.94; 17–54); 99.70% (*N* = 662) were Caucasian. Regarding marital status, 85.39% were unmarried (*N* = 567), 13.55% married (*N* = 90) and 0.90% (N = 6) divorced (1 unkown).

The mean BMI was 22.24 kg/m^2^ (SD: 3.66; min 15.78, max 39.06). Sport activity was practiced by 56.93% of the students (*N* = 378); dieting and food supplements use were reported by 12.20% (*N* = 81) and 16.42% (*N* = 109) of the sample. Current and past EDs were self-reported by 6.48% (*N* = 43), 3.01% (*N* = 20) of the students, respectively.

Regarding questionnaire scores in the whole sample, the mean EAT-26 scores were 5.04 ± 6.06 for Dieting, 1.78 ± 2.93 for Oral Control, 0.99 ± 2.23 for Bulimia and Food Preoccupation; the total EAT-26 score was 7.89 ± 9.51. ORTO-15 and EAT-26 scores dichotomized according to scoring below or above the respective cutoff, and TCI scores classified in low, medium, high according to the mean value of each dimension (as described in the methods section) are reported in Table 1 for the whole sample.

**Table 1:** ORTO-15, EAT-26 and TCI in the whole sample (Italian, Polish and Spanish students)

Questionnaire	N	%
ORTO 15		
Score < cut-off	246	37.05
Score > cut-off	418	62.95
EAT-26		
Score ≥ 20	56	8.43
Score < 20	608	91.57
TCI		
Novelty Seeking (mean score)		
Low/very low	137	20.63
Medium-low/medium/medium-high	332	50.00
High/very high	183	27.56
Missing	12	1.81
Harm Avoidance (mean score)		
Low/very low	69	10.39
Medium-low/medium/medium-high	332	50.00
High/very high	250	37.65
Missing	13	1.96
Reward Dependence (mean score)		
Low/very low	247	37.20
Medium-low/medium/medium-high	306	46.08
High/very high	98	14.76
Missing	13	1.96
Persistence (mean score)		
Low/very low	252	37.95
Medium-low/medium/medium-high	293	44.13
High/very high	106	15.96
Missing	13	1.96
Self-Directedness (mean score)		
Low/very low	288	43.37
Medium-low/medium/medium-high	296	44.58
High/very high	69	10.39
Missing	11	1.66
Cooperativeness (mean score)		
Low/very low	325	48.95
Medium-low/medium/medium-high	259	39.01
High/very high	69	10.39
Missing	11	1.66
Self-Transcendence (mean score)		
Low/very low	361	54.37
Medium-low/medium/medium-high	246	37.05
High/very high	43	6.48
Missing	14	2.11

### Univariable and multivariable logistic regression: associations between ORTO-15 scores, the variables assessed and questionnaire scores (EAT-26, TCI)

The results of the univariable and multivariable logistic regression analyses performed to assess the association of ON (as described by an ORTO 15 score below the cutoff; dependent variable in the multivariable analysis) with the self-report variables gathered from participants and questionnaire scores (EAT-26 and TCI) (independent variables in the multivariable analysis) are reported in Table 2 and Table 3, respectively.

**Table 2:** Univariable analysis: associations between ON (ORTO-15 score < cutoff), the self-report variables assessed, EAT-26 and TCI scores

ORTO-15 < cutoff (ORTO-15 > cut-off reference)	N	Odds ratio	95% Confidence Interval	*p*-value
Females	663	1.71	1.18–2.47	**0.004**
Age	664	0.99	0.96–1.03	0.724
Marital Status, Unmarried	663	1.21	0.76–1.94	0.426
BMI	659	1.05	1.00–1.09	**0.040**
No Sport Activity	664	1.24	0.90–1.70	0.192
Dieting	664	2.67	1.66–4.28	**0.000**
Food Supplements	664	1.42	0.94–2.15	0.099
Self-report current ED	664	3.10	1.63–5.87	**0.001**
No self-report past ED	664	0.58	0.24–1.41	0.229
EAT-26				
Score ≥ 20	664	2.89	1.65–5.06	**0.000**
TCI^%^				
NS low and medium	652	0.94	0.66–1.34	0.746
HA low and medium	651	1.03	0.74–1.43	0.846
RD low and medium	651	1.48	0.93–2.36	0.100
P low and medium	651	2.13	1.32–3.44	**0.002**
SD low and medium	653	1.05	0.62–1.76	0.859
C low and medium	653	1.13	0.67–1.90	0.659
ST low and medium	650	1.55	0.78–3.09	0.208

= reference: high

*P*-values set in boldface indicate statistical significance

**Table 3:** Multivariable logistic regression model for variables predicting ON (ORTO-15 score < cutoff) (only statistically significant results are shown)

ORTO-15 < cutoff (ORTO-15: > cut-off reference)	N	Odds ratio	95% Confidence Interval	p-value
Female	650	1.52	1.03–2.25	0.036
Dieting	650	2.52	1.55–4.10	0.000
P low and medium	650	1.95	1.19–3.19	0.008

Details about the descriptive data of the self-report variables and questionnaire scores with a statistically significant association with the ORTO-15 score are reported in Table 4.

**Table 4:** Descriptive data of the statistically significant associations between ORTO-15 score (> or < cutoff), self-report variables and questionnaire scores in the whole sample

	ORTO-15 score > cutoff % (N)	ORTO-15 score < cutoff % (N)
	62.95 (418)	37.05 (246)
Gender		
Male	31.34 (131) 71.58	21.14 (52) 28.42
Female	68.42 (286) 59.58	78.86 (194) 40.42
Missing	0.24 (1) 100	0.00 0.00
Dieting		
YES	8.13 (34) 41.98	19.11 (47) 58.02
NO	91.87 (384) 65.87	80.89 (199) 34.13
Self-reported current ED		
YES	3.83 (16) 37.21	10.98 (27) 62.79
NO	96.17 (402) 64.73	89.02 (219) 35.27
EAT-26		
Score ≥ 20	5.26 (22) 39.29	13.82 (34) 60.71
Score < 20	94.74 (396) 65.13	86.18 (212) 34.87
TCI Persistence		
Low and medium mean score	78.71 (329) 60.37	87.80 (216) 39.63
High mean score	19.38 (81) 76.42	10.16 (25) 23.58
Missing	1.91 (8) 61.54	2.03 (5) 38.46

### Comparison among the Italian, Polish and Spanish samples

The results of the statistical analyses performed to compare the Italian, Polish and Spanish samples are reported in Table 5. While no statistically significant result was found from the chi-suqare comparison of those scoring higher/lower that the EAT-26 cutoff, the Kruskal-Wallis comparison of the EAT-26 total mean scores in the three countries yielded the following result: it was higher in the Italian than in the Polish and Spanish Sample (9.56 ± 12.28; 8.52 ± 8.18; 5.87 ± 7.11; *p* = 0.0001).

**Table 5:** Comparison among the Italian, Polish and Spanish sample: socio-demographic, clinical information, EAT-26 and ORTO-15 questionnaires scores

	Italian sample % (N)	Polish sample % (N)	Spanish sample % (N)	P
	32.53 (216)	31.02 (206)	36.45 (242)	
Gender				
Males	26.77 (34) 22.97	12.14 (25) 16.89	36.78 (89) 60.14	**< 0.0001**
Females	73.23 (93) 21.78	87.86 (181) 42.39	63.22 (153) 35.83	
Sport Activity				
Yes	63.43 (137) 36.24	47.57 (98) 25.93	59.09 (143) 37.83	**0.003**
No	36.57 (79) 27.62	52.43 (108) 37.76	40.91 (99) 34.62	
Dieting				
Yes	1.85 (4) 4.94	21.84 (45) 55.56	13.22 (32) 39.51	**< 0.0001**
No	98.15 (212) 36.36	78.16 (161) 27.62	86.78 (210) 36.02	
Food Supplements				
Yes	19.91 (43) 39.45	25.24 (52) 47.71	5.79 (14) 12.84	**< 0.0001**
No	80.09 (173) 31.17	74.76 (154) 27.75	94.21 (228) 41.08	
Self-reported current ED				
Yes	0.00 (0) 0.00	20.87 (43) 100.00	0.00 (0) 0.00	**< 0.0001**
No	100.00 (216) 34.78	79.13 (163) 26.25	100.00 (242) 38.97	
Self-reported past ED				
Yes	2.31 (5) 25.00	5.83 (12) 60.00	1.24 (3) 15.00	**0.02**
No	97.69 (211) 32.76	94.17 (194) 30.12	98.76 (239) 37.11	
ORTO-15				
Score < cut-off	30.09 (65) 26.42	66.50 (137) 55.69	18.18 (44) 17.89	**< 0.0001**
Score > cut-off	69.91 (151) 36.12	33.50 (69) 16.51	81.82 (198) 47.37	
EAT-26				
Score ≥ 20	7.87 (17) 30.36	11.17 (23) 41.07	6.61 (16) 28.57	0.21
Score < 20	92.13 (199) 32.73	88.83 (183) 30.10	93.39 (226) 37.17	

*P*-values set in boldface indicate statistical significance

## Discussion

### Descriptive analysis

The first aim of the present study was to investigate the prevalence of ON (as suggested by an ORTO-15 score below the cutoff) in a sample composed by 664 university students enrolled in Italy (*N* = 216), Poland (*N* = 206) and Spain (*N* = 242).

The ORTO-15 score suggested ON in more than a third of the whole sample of students (score < cutoff in 37.05%). The quite high value we found is consistent with reports from previous studies with the same assessment tool, which reported a prevalence of ON up to more than 50% in university students from different countries [41–43]. More specifically, some at-risk groups have been described, including students attending university courses focused on nutrition (Dietetics) [44, 45] and body care (Exercise and Sport Sciences) [45]), as well as in medical students and residents [32, 46].

Anyway, the interpretation of the result about ON prevalence is currently difficult, because ON is not a clinical diagnosis and the ORTO-15 is far from being a diagnostic tool. Moreover, the possible implications of ON for the general population still need to be better understood (for instance, eating healthy may be desirable until it does not become clinically impairing).

Overall, although more studies on ON are required, in our sample the prevalence of ON, self-reported current/previous EDs (6.48 and 3.01%, respectively), and the percentage of students scoring above the EAT-26 cutoff (suggestive of high-risk for EDs, 8.43%), suggest that attention should be paid to eating-related problems in university students, and that psychoeducational interventions targeting this topic may be warranted also for this population [47].

### Associations between orthorexia nervosa, the variables assessed and questionnaire scores (EAT-26, TCI)

The second objective was to investigate the association between ON, socio-demographic features, eating patterns (measured with the EAT-26) and personality traits (assessed with the TCI) within the whole sample.

The univariable analyses shown an increased risk of ON in female students, with higher BMI values, self-reporting themselves as dieting and having a current ED, and in those scoring above the EAT-26 cutoff.

The multivariables analyses confirmed an increased risk of ON in females and in students self-reporting themselves as dieting. An increased likelihood of ON was found also in students with low or medium Persistence (as measured by the TCI).

Regarding gender, while it is widely acknowledged that the prevalence of EDs like AN has a male to female ratio 1:9 [48], gender differences in ON are still a matter of debate; results are inconsistent across studies [9, 49], and some did not even show any significant gender difference [25, 32, 42].

Literature findings are inconsistent also about the relationship between ON and BMI. In the current research, the likelihood of ON increased with higher BMI values. Some studies have not found any significant relationship, while others described an increasing trend of ON with increasing BMI values [32]; no study has found yet a negative correlation between BMI and orthorexia. Nonetheless, the low OR does not allow to draw clear conclusions about the clinical relevance of this association.

Students who declared to be dieting were more likely to display ON than those who were not following a diet. ON has already been described as being more frequent in individuals who strictly adhere to dieting habits, but once again results are inconsistent and another study [50] failed to identify any correlation between being dieting and ON.

Last, there was a higher risk of ON in students who self-reported having a current ED at the time of assessment, but not in those with a self-reported history of EDs. Furthermore, in the current study we also found a positive correlation between ON and high risk of EDs as suggested by an EAT-26 score above the cutoff.

The relationship between ON and EDs is complex and still far from being thoroughly understood. Afive-fold increase in the risk of having ON was identified in individuals with a disordered eating behavior compared to those with normal eating attitudes [21], and a frequent comorbidity, either current or lifetime (from 28 to 53% in a 3-year follow-up study), has been described between ON and EDs [35, 36]. Orthorexia can precede the onset of an ED, or it can represent its evolution in the phase of remission and recovery, representing a condition with the “advantage” of making the invidual with an ED feels once again accepted and part of society; this is one reason why ON can be described as “a disease masked by virtue” [3].

Moreover, EDs have been identified as risk factors for ON by some authors [34], while others have found EDs and weight concern as negative predictors of ON [36, 51]. Also, we cannot exclude that in some cases inconsistent results in the literature could be due to the use of different instruments, which may likely measure different facets of orthorexia [52].

Despite the intrinsic limitations of self-report information about previous/current ED, and those retrieved from the EAT-26 scoring, overall, from our results described above and from the inconsistency of those available in the existing literature, it emerges the importance of fully defining the construct and implications of ON, and its possible relation with EDs, in order to better understand how to approach it in a more scientifically sound way.

An interesting result was found regarding the correlation between ON and personality features as assessed by the TCI. ON was more likely in students with low and medium scores on the P scale of the TCI than in those with high P scores. Persistence reflects the capacity for perseveration, determination and constancy in spite of frustration and fatigue, and predicts resistance to the extinction of behavior in the face of intermittent reinforcements [19]; it is usually characterized by high scores in typical descriptions of AN, restricter sybtype [53]. To date, only one study has investigated personality traits in ON with the TCI [24], finding a profile characterized by high HA, low SD and high ST, while no significant result emerged regarding P. The “orthorexic personality” has been previously described as characterized by an excessive preoccupation that shows itself with shyness in social situations, combined with the desire to be perfect and to feel accepted. In this context, “pure” dieting would result from an intense need for control to compensate for low self-esteem, feelings of ineffectiveness and inability to manage harmful events [18]. The low P we found in ON subjects may reflect a poor ability in adopting appropriate coping strategies to finalize action in everyday life. While this result, again, is difficult to contextualize given the shortage of available studies in the literature, further research about the personality features of ON individuals are warranted to identify possible targets for psychoeducational and also therapeutic interventions (such as perfectionism, need for control, etc.).

### Comparison among the Italian, Polish and Spanish samples

The third aim of our study was to highlight any cross-cultural difference in eating attitudes (as measured by the EAT-26) and ON in the three sub-groups of students (notwithstanding the limitations of the comparability of the different university populations recruited in the three countries due to the convenience sampling procedure).

Differences among the three subgroups were found for all the self-reported information we gathered. While less Polish students reported practicing physical/sport activity, they were more frequently dieting and using food supplements (e.g. vitamins), compared to their peers in Italy and Spain.

A few studies are available about eating habits in Polish university students [54, 55]; with more detail, a recent research found almost a third of the studied population following restrictions in the type and amount of food intake [56], consistent with our findings on dieting.

To contextualize these results (dieting, use of food supplements), it should be considered that in Poland, in recent decades, economic and political changes have had a significant impact on the lifestyle of various social groups, especially on young people, including university students [56].

Regarding self-reported current and past ED, the only students declaring a current ED belonged to the Polish sample, and the higher rate of students with a self-reported history of ED was found in the Polish sample, as well, even though no actual difference in the BMI of students belonging to the three subgroups emerged. While all this information is intrinsically limited by its self-report nature, we cannot exclude that a different perception of EDs may underlie these findings.

The opposite trend was found for sport activity practiced by students in the three subgroups, which was less frequent in the Polish sample (only in the Polish sample the percentage of students who did not practice sports was more than 50%). Nonetheless, another study, involving a different population of university students (from medical/scientific studies rather than humanities faculties as in the current one) showed that the majority of Polish students rated their activity level as medium (68.5%) and high (25.3%) and that 80.5% of female students and 74.3% of men practiced active sports every day [54].

Despite inconsistencies and gaps in the available literature, and the limitations of our results, it will certainly be interesting to better understand whether cultural background differences among the three countries exist in terms of what students consider a healthy lifestyle and in how they believe it can be achieved.

To our knowledge, in the last years in Poland good consumer practices have gained greater popularity (for example in the control of the composition of food products) and healthy eating has become the most important among the strategies aimed at improving one’s state of health [57].

On the other hand, Italy has a widely recognized culture about eating and nutrition and the Mediterranean diet has long been praised for its general health benefits and a strong attention paid to taste [58]; a similar situation is found in the Northeast (Basque Country) region of Spain.

Good consumer practices, control of the composition of food products and the belief that healthy dieting is the most important approach for the improvement of ones’ health could explain the high percentage of ON found in the Polish sample. On the other hand, it is likely that the lowest frequency of ON and attitudes found in the Spanish sample depends on an enogastronomic culture that includes the Mediterranean diet style, similar to the Italian one, but with an even stronger convivial and social value of the meal.

Moreover, since the importance attributed to conviviality and to the eating-related social dimension could penalize orthorexic individauls, we cannot exclude that our results depend on under-reporting on behalf of students. A clearer definition of ON and its diagnostic criteria, and further studies using clinical interview and assessment rather than self-report information will allow a more comprehensive approach to the intriguing topic of the impact of socio-cultural differences on the ON construct.

### Limitations

Some limitations of the current study should be underscored. As already stated, an assessment based on self-administered questionnaires entails problems in terms of realibility and possible underestimation/overestimation. The ORTO-15 Questionnaire does not allow to make a diagnosis of ON, and has intrinsic limitations already described by the scientific literature [59], with contradictory results regarding its psychometric properties, including construct validity [9, 60–63].

The possible problems due to the convenience sampling procedure and to different cut-offs and scoring methods of the scales used in the three countries have been overcome as described above in the methods section.

On the other hand, a strength of the study is that it represents the first one in the scientific literature specifically focused on the comparison of three countries as far as ON and eating patterns are concerned.

## Conclusions

The positive association between ON, a self-reported current ED, presence of food psychopathology as suggested by the EAT-26 score, BMI and adherence to a dieting that emerged in the present study needs to be supported by further research in order to better understand the relationship between ON and ED. The current knowledge suggests that ON tendencies are observed in those who expect benefits for their health, society and the environment, deriving from food, but may also be found in individuals who may rather try to disguise disordered eating attitudes with a more socially acceptable eating habit [8, 19, 64].

Regarding the differences in the frequency of ON found among the Italian, Polish and Spanish samples, we cannot exclude that a role is played by gastronomic culture, Mediterranean diet, convivial and social value attributed to eating and main approaches aimed at improving one’s health typical of each socio-cultural context.

Further studies with consistent and sound methodological approaches will help to shed light on the several gaps that still exist in this field of research, including that about whether ON is mediated by socio-cultural factors and to what extent.

## Data Availability

The datasets used and/or analysed during the current study are available from the corresponding author on reasonable request.

## Abbreviations

AN: Anorexia Nervosa
ARFID: Avoidant/Restrictive Food Intake Disorder
BMI: Body Mass Index
C: Cooperativeness
DSM-5: Diagnostic and Statistical Manual of Mental Disorders
EAT-26: Eating Attitudes Test-26
ED: Eating Disorder
HA: Harm Avoidance
NS: Novelty Seeking
OCD: Obsessive Compulsive Disorder
ON: Orthorexia nervosa
P: Persistence
RD: Reward Dependence
SD: Self-Directedness
ST: Self-Transcendence
TCI: Temperament and Character Inventory

## References

[1] Healy D. From fasting saints to anorexic girls: the history of self-starvation. Walter Vandereycken, Ron van Deth. London: Athlone Press, 1994. Irish Journal of Psychological Medicine. Cambridge University Press; 1995;12(1):41–2.

[2] Bundros J, Clifford D, Silliman K, Morris MN (2016). Prevalence of orthorexia nervosa among college students based on Bratman’s test and associated tendencies. Appetite..

[3] Daniele MT, Pinto M, Manna V. Il cibo come droga Un approccio integrato ai disturbi del comportamento alimentare nel XXI secolo. Collana di Psicoterapia e Psicoanalisi Edizioni Alpes. 2016:85–95.

[4] Crawford R (1980). Healthism and the medicalization of everyday life. Int J Health Serv.

[5] Håman L, Barker-Ruchti N, Patriksson G (2015). Lindgren E-C. Orthorexia nervosa: An integrative literature review of a lifestyle syndrome Int J Qual Stud Health Well-Being.

[6] Abbate-Daga G, Marzola E, Gramaglia C, Brustolin A, Campisi S, De-Bacco C, Amianto F, Fassino S (2012). Emotions in eating disorders: changes of anger control after an emotion-focused day hospital treatment. Eur Eat Disord Rev.

[7] Dunn Thomas M., Bratman Steven (2016). On orthorexia nervosa: A review of the literature and proposed diagnostic criteria. Eating Behaviors.

[8] Koven NS, Abry AW (2015). The clinical basis of orthorexia nervosa: emerging perspective. Neuropsychiatry Dis Treat.

[9] Moroze RM, Dunn TM, Holland JC, Yager J, Weintraub P (2014). Microthinking about micronutrients: a case of transition from obsession about healthy eating to near-fatal "orthorexia nervosa" and proposed diagnostic criteria. Psychosomatics..

[10] Barthels F, Meyer F, Huber T, Pietrowsky R (2017). Orthorexic eating behaviour as a coping strategy in patients with anorexia nervosa. Eat Weight Disord.

[11] Barthels F, Meyer F, Pietrowsky R (2015). Orthorexic eating behavior. A new type of disordered eating. Ernahrungs Umschau.

[12] Cuzzolaro M, Donini LM (2016). Orthorexia nervosa by proxy?. Eat Weight Disord.

[13] Barnes MA (2017). Caltabiano ML. The interrelationship between orthorexia nervosa, perfectionism, body image and attachment style Eat Weight Disord.

[14] Dell’Osso L, Abelli M, Carpita B, Pini S, Castellini G, Carmassi C, Ricca V (2016). Historical evolution of the concept of anorexia nervosa and relationships with orthorexia nervosa, autism, and obsessive–compulsive spectrum. Neuropsychiatr Dis Treat.

[15] Forester DS (2014). Examining the relationship between orthorexia nervosa and personality traits (unpublished master's thesis).

[16] Oberle CD, Samaghabadi RO, Hughes EM (2017). Orthorexia nervosa: assessment and correlates with gender, BMI, and personality. Appetite..

[17] Kiss-Leizer M, Rigo A (2018). People behind unhealthy obsession to healthy food: the personality profile of tendency to orthorexia nervosa. Eat Weight Disord.

[18] Cloninger CR, Przybeck TR, Svrakic DM, Wetzei RB (1994). The temperament and Character Inventory (TCI): a guide to its developement and use. Center for Psychobiology of personality.

[19] Gramaglia C, Brytek-Matera A, Rogoza R, Zeppegno P (2017). Orthorexia and anorexia nervosa: two distinct phenomena? A cross-cultural comparison of orthorexic behaviours in clinical and non-clinical samples. BMC Psychiatry.

[20] Sanlier N, Yassibas E, Bilici S, Sahin G, Celik B (2016). Does the rise in eating disorders lead to increasing risk of orthorexia nervosa? Correlations with gender, education, and body mass index. Ecol Food Nutr.

[21] Scarff JR (2017). Orthorexia nervosa: an obsession with healthy eating. Fed Pract.

[22] Gaudio S, Dakanalis A (2018). Personality and eating and weight disorders: an open research challenge. Eat Weight Disord.

[23] Fassino S, Amianto F, Gramaglia C, Facchini F, Abbate DG (2004). Temperament and character in eating disorders: ten years of studies. Eat Weight Disord.

[24] Brytek-Matera A, Donini LM, Krupa M, Poggiogalle E, Hay P. Orthorexia nervosa and self-attitudinal aspects of body image in female and male university students. J Eat Disord. 2015; 24; 3:2. 10.1186/s40337-015-0038-2 PMC4359442 25774296

[25] Donini LM, Marsili D, Graziani MP, Imbriale M, Cannella C (2005). Orthorexia nervosa: Validation of a diagnosis questionnaire. Eat Weight Disord.

[26] Brytek-Matera A, Fonte ML, Poggiogalle E, Donini LM, Cena H (2017). Orthorexia nervosa: relationship with obsessive-compulsive symptoms, disordered eating patterns and body uneasiness among Italian university students. Eating Weight Disord.

[27] Garner DM, Olmsted MP, Bohr Y, Garfinkel PE (1982). The eating attitudes test: psychometric features and clinical correlates. Psychol Med.

[28] Gutiérrez-Zotes JA, Bayón C, Montserrat C, Valero J, Labad A, Cloninger CR, Fernández-Aranda F (2004). Inventario del Temperamento y el Carácter-Revisado (TCI-R). Baremación y datos normativos en una muestra de población general. Actas Esp Psiquiatr.

[29] Heiss S, Coffino JA, Hormes JM (2019). What does the ORTO-15 measure? Assessing the construct validity of a common orthorexia nervosa questionnaire in a meat avoiding sample. Appetite..

[30] Bratman S (2017). Orthorexia vs theories of healthy eating. Eat Weight Disord.

[31] Aksoydan E, Camci N (2009). Prevalence of orthorexia nervosa among turkish performance artists. Eat Weight Disord.

[32] Fidan T, Ertekin V, Işikay S, Kirpinar I (2010). Prevalence of orthorexia among medical students in Erzurum. Turkey Compr Psychiatry.

[33] Varga M, Dukay-Szabó S, Túry F, van Furth EF (2013). Evidence and gaps in the literature on orthorexia nervosa. Eat Weight Disord.

[34] Segura-Garcia C, Ramacciotti C, Rania M, Aloi M, Caroleo M, Bruni A, Gazzarrini D, Sinopoli F, De Fazio P (2015). The prevalence of orthorexia nervosa among eating disorder patients after treatment. Eat Weight Disord.

[35] Brytek-Matera A, Krupa M, Poggiogalle E, Donini LM (2014). Adaptation of the ORTHO-15 test to polish women and men. Eat Weight Disord.

[36] Dotti A, Lazzari R (1998). Validation and reliability of the Italian EAT-26. Eat Weight Disord.

[37] Rogoza Radosław, Brytek-Matera Anna, Garner David (2016). Analysis of the EAT-26 in a non-clinical sample. Archives of Psychiatry and Psychotherapy.

[38] Castro J, Toro J, Salamero M, Guimerá E (1991). The eating attitudes test: validation of the Spanish version. Evaluación Psicológica.

[39] Vespa A, Ottaviani M, Fossati A, Giulietti MV, Spatuzzi R, Meloni C, Fabbietti P, Spazzafumo L, Rozsa S, Cloninger RC (2015). Validation of the Italian translation of the revised temperament and character inventory--TCI-140--in adult participants and in participants with medical diseases. Compr Psychiatry.

[40] Zakrzewska M, Samochowiec J, Rybakowski F, Hauser J, Pełka-Wysiecka J (2001). Polska wersja Inwentarza Temperamentu i Charakteru (TCI): analiza rzetelności. Psych Pol.

[41] Brytek-Matera A, Gramaglia C, Zeppegno P (2014). Healthy eating obsession in women with anorexia nervosa: a case control study. New developments in anorexia nervosa research.

[42] Ramacciotti CE, Perrone P, Coli E, Burgalassi A, Conversano C, Massimetti G, Dell’Osso L (2011). Orthorexia nervosa in the general population: a preliminary screening using a self-administered questionnaire (ORTO-15). Eat Weight Disord.

[43] Jahrami H, Sater M, Abdulla A, Faris MA, AlAnsari A. Eating disorders risk among medical students: a global systematic review and meta-analysis. Eat Weight Disord. 2018 May;21. 10.1007/s40519-018-0516-z 29785631

[44] Kinzl JF, Hauer K, Traweger C, Kiefer I (2006). Orthorexia nervosa in dieticians. Psychother Psychosom.

[45] Bo S, Zoccali R, Ponzo V, Soldati L, De Carli L, Benso A, Fea E, Rainoldi A, Durazzo M, Fassino S, Abbate-Daga G. University courses, eating problems and muscle dysmorphia: are there any associations? J Transl Med. 2014 7;12:221. 10.1186/s12967-014-0221-2 PMC4256707 25095736

[46] Bağci Bosi AT, Camur D, Güler C (2007). Prevalence of orthorexia nervosa in resident medical doctors in the faculty of medicine (Ankara, Turkey). Appetite..

[47] Yager Z, O’Dea JA (2008). Prevention programs for body image and eating disorders on University campuses: a review of large, controlled interventions. Health Promot Int [Internet].

[48] Keller MF, Konradsen H (2013). Orthorexia in young fitness participants. Klinisk Sygepleje.

[49] Karakus B, Hidiroglu S, Keskin N, Karavus M (2017). Orthorexia nervosa tendency among students of the department of nutrition and dietetics at a university in Istanbul. North Clin Istanb.

[50] McInerney-Ernst EM (2011). Orthorexia nervosa: real construct or newest social trend?.

[51] Barrada JR, Roncero M. Bidimensional Structure of the Orthorexia: Development and Initial Validation of a New Instrument. analesps [Internet]. 10Apr.2018 [cited 13Apr.2019];34(2):283–291.

[52] Fassino S, Pierò A, Levi M, Gramaglia C, Amianto F, Leombruni P, Abbate Daga G (2004). Psychological treatment of eating disorders. A review of the literature. Panminerva Med.

[53] Jakubiec D, Kornafel D, Cygan A, Górska-Kłęk L, Chromik K (2015). Lifestyle of students from different universities in Wroclaw. Poland Rocz Panstw Zakl Hig.

[54] El Ansari W, Stock C, Mikolajczyk RT (2012). Relationships between food consumption and living arrangements among university students in four European countries - A cross-sectional study. Nutr J [Internet].

[55] Galinski G, Lonnie M, Kowalkowska J (2016). Self-reported dietary restrictions and dietary patterns in polish girls: a short research report (GEBaHealth study). Nutrients..

[56] CBOS (Centrum Badania Opinii Społecznej). Zachowania żywieniowe Polaków. Nr 115/2014. Warszawa; 2014. http://www.cbos.pl/SPISKOM.POL/2014/K_115_14.PDF.

[57] Schwingshackl L, Missbach B, König J (2015). Hoffmann G adherence to a Mediterranean diet and risk of diabetes: a systematic review and meta-analysis. Public Health Nutr.

[58] Missbach B, Hinterbuchinger B, Dreiseitl V, Zellhofer S, Kurz C, König J (2015). When eating right, is measured wrong! A validation and critical examination of the ORTO-15 questionnaire in German. PLoS One.

[59] Missbach B, Dunn TM, König JS. We need new tools to assess Orthorexia Nervosa A commentary on “Prevalence of Orthorexia Nervosa among College Students Based on Bratman’s Test and Associated Tendencies Appetite. 2017; 108:521–524. 10.1016/j.appet.2016.07.010 27401768

[60] Cena H, Barthels F, Cuzzolaro M, Bratman S, Brytek-Matera A, Dunn T, Varga M, Missbach B, Donini LM. Definition and diagnostic criteria for orthorexia nervosa: a narrative review of the literature. Eat Weight Disord. 2019;24(2):209-46. 10.1007/s40519-018-0606-y. 10.1007/s40519-018-0606-y 30414078

[61] Moller S, Apputhurai P, Knowles SR (2019). Confirmatory factor analyses of the ORTO 15-, 11- and 9-item scales and recommendations for suggested cut-off scores. Eat Weight Disord.

[62] Roncero M, Barrada JR, Perpiñá C (2017). Measuring orthorexia nervosa: psychometric limitations of the ORTO-15. Span J Psychol.

[63] Brytek-Matera A, Gramaglia CM, Gambaro E, Delicato C, Zeppegno P (2018). The psychopathology of body image in orthorexia nervosa. Journal of Psychopatology.

[64] Rich E, Holroyd R, Evans J, Evans J, Davies B, Wright J (2004). Hungry to be noticed: young women, anorexia and schooling.

